# NO *Candida auris*: Nitric Oxide in Nanotherapeutics to Combat Emerging Fungal Pathogen *Candida auris*

**DOI:** 10.3390/jof6020085

**Published:** 2020-06-12

**Authors:** Levi G. Cleare, Kevin L. Li, Waleed M. Abuzeid, Parimala Nacharaju, Joel M. Friedman, Joshua D. Nosanchuk

**Affiliations:** 1 Department of Medicine (Division of Infectious Diseases) and Department of Microbiology and Immunology, Albert Einstein College of Medicine, Bronx, NY 10461, USA; levi.cleare@einsteinmed.org; 2 Department of Otorhinolaryngology–Head and Neck Surgery, Albert Einstein College of Medicine, Bronx, NY 10461 USA; kevin.li@einsteinmed.org; 3 Department of Otolaryngology: Head and Neck Surgery, University of Washington, Seattle, WA 98195, USA; wabuzeid@uw.edu; 4 Department of Physiology and Biophysics, Albert Einstein College of Medicine, Bronx, NY 10461, USA; parimala.nacharaju@einsteinmed.org (P.N.); joel.friedman@einsteinmed.org (J.M.F.)

**Keywords:** *Candida auris*, C. auris, nitric oxide, NAC, nanoparticles, confocal microscopy

## Abstract

*Candida auris* (*C. auris*) is an emerging pathogenic fungal species that is especially worrisome due to its high mortality rates and widespread antifungal resistance. Previous studies have demonstrated the efficacy of nitric oxide (NO) nanoparticles on *Candida* species, and, to our knowledge, this is the first study to investigate the antifungal effects of a NO-generating nanoparticle on *C. auris*. Six *C. auris* strains were incubated with a nanoparticle (NAC-SNO-np), which releases *N*-acetylcysteine *S*-nitrosothiol (NAC-SNO) and *N*-acetylcysteine (NAC), and generates NO, through colony forming unit (CFU) assays, and confocal laser scanning microscopy. NAC-SNO-np effectively eradicates planktonic and biofilm *C. auris*. Across all six strains, 10 mg/mL NAC-SNO-np significantly reduced the number of CFUs (*p* < 0.05) and demonstrated a >70% decrease in biofilm viability (*p* < 0.05). NAC-SNO-np effectively eradicates planktonic *C. auris* and significantly reduces *C. auris* biofilm formation. Hence, this novel NO-releasing nanoparticle shows promise as a future therapeutic.

## 1. Introduction

*Candida auris* (*C. auris*) is an emerging pathogenic fungal species first isolated in Japan in 2009 [1]. *C. auris* simultaneously emerged on three separate continents and is notable in that isolates from each continent are from different clades [2]. *C. auris* has now spread to all continents except for Antarctica [3,4,5,6,7,8,9]. *C. auris* is phylogenetically most closely related to *C. haemulonii*. This phylogenetic relatedness has made the actual global distribution unclear, in part due to the inability to properly identify *C. auris* using classic microbiological techniques, commonly misidentifying it as the closely related *C. haemulonii* [10,11,12]. While the exact global distribution remains unclear, it is apparent that *C. auris* is quickly becoming more prevalent.

*C. auris* can colonize multiple body areas, ranging from the ears and nares to the groin and rectum. It can be acquired from contact with patients harboring *C. auris*. Indeed, Horton et al. demonstrated recently that *C. auris* formed biofilms in both synthetic sweat media, mimicking the physiologic conditions of the axilla, and on porcine skin [13]. Much more worrisome is acquisition from contact with contaminated surfaces with *C. auris* biofilms having been shown to survive simulated desiccation [13]. Reports of *C. auris* outbreaks in the United States show that these infections likely began in health care facilities and were transmitted nosocomially [14,15]. This is particularly concerning as *C. auris* harbors a predilection for causing severe candidemia in critically ill patients [6,14,16,17]. *C. auris* has an unclear mortality rate, with wide variation based on region and underlying comorbidities. In the United States, 30 day mortality rates related to candidemia exceed 50% [14,16,18]. 

*C. auris* exhibits widespread resistance to antifungal agents. Lockhart et al. examined resistance rates in isolates from 54 patients representing the different clades and found that 93% of isolates were resistant to fluconazole, 35% to amphotericin B, and 7% to echinocandins [2]. This resistance profile has prompted physicians to prescribe echinocandins empirically for suspected *C. auris* infections [19]. It is further notable that 41% of isolates were resistant to two separate antifungal classes and 4% were resistant to all three antifungal classes [2,20]. In light of the widespread antifungal resistance and significant morbidity and mortality rates, it is imperative to develop novel approaches for combatting *C. auris*. 

Nitric oxide (NO) is an endogenously generated diatomic gas that is important for cellular signaling, vascular modulation, and immune function, and is a vital component of mammalian host defenses [21]. NO is an innate immune system product and acts as both a cytostatic and cytotoxic broad-spectrum antimicrobial agent. NO has antimicrobial properties against many bacteria, yeasts, helminths, protozoa, and viruses [22]. Notably, in terms of fungi, NO has previously demonstrated antimicrobial efficacy against *C. albicans* [22,23,24]. Moreover, we have developed a NO nanoparticle delivery platform that has demonstrated antimicrobial efficacy against *C. albicans* through disruption of fungal growth and morphogenesis [25,26,27]. The NO nanoparticles can kill both hyphae and yeast forms within mature biofilms, predominantly by inducing apoptosis and subsequent cell necrosis [25]. Recently, Vargas-Cruz et al. demonstrated the efficacy of a nitroglycerin-citrate-ethanol catheter lock solution, which converts nitroglycerin into NO, in the eradication of *C. auris* biofilms in central line lumens. The authors further showed that the biofilm eradication was superior to common antifungal medications such as amphotericin B, fluconazole, and caspofungin, among others, in vitro [28]. 

In this study, we utilized a novel nanoparticle (NAC-SNO-np), which releases *N*-acetylcysteine *S*-nitrosothiol (NAC-SNO) to induce a sustained release of NO and explored the efficacy of NAC-SNO-np in disrupting *C. auris.* To our knowledge, this is the first study to investigate the antifungal effects of NO on both planktonic and biofilm forms of *C. auris*.

## 2. Materials and Methods

### 2.1. Candida auris Strains

A total of six *C. auris* strains were used in this study. Two clinical strains (MMC 1 and MMC 2) were isolated at the Montefiore Medical Center (MMC; Bronx, NY, USA) and we have previously characterized their resistance phenotypes and multi-omics signatures [29]. The samples were obtained with the written consent of all patients under a protocol that was approved by the institutional review boards (IRB Number: 2016–7455 approved on 2 July 2017) of the Albert Einstein College of Medicine and MMC. Four reference strains (CDC 0381, CDC 0383, CDC 0385, and CDC 0388), from major clades around the world, were obtained from the *C. auris* panel of the Centers for Disease Control (CDC; Atlanta, GA, USA) and Food and Drug Administration (FDA; Silver Spring, MD, USA) Antibiotic Resistance Isolate Bank (https://www.cdc.gov/drugresistance/resistance-bank/). Minimal Inhibitory Concentrations (MICs) for *C. auris* to amphotericin B, fluconazole and echinocandins from MMC [29], and the CDC have been previously described. Hence, the strains selected represent both susceptible and resistant isolates. All strains were stored in liquid nitrogen and, suspended in Sabouraud (SAB) broth with 50% glycerol, until use. Strains were grown in SAB broth for 24 h at 37 °C using a rotary shaker set at 150 rpm.

### 2.2. C. auris Resistance Profiles

Six diverse *C. auris* strains, clinical and reference, were used to examine the generalizability of the efficacy of the NAC-SNO-np against this species. These strains included the original Japanese strain and encompass four separate continents. Three of our six strains were resistant to fluconazole, and one strain (CDC 0383) was resistant to both fluconazole and caspofungin. No strain in our study was resistant to amphotericin B (Table 1).

### 2.3. Synthesis of NAC-SNO-np

#### 2.3.1. Preparation of Sol-Gel

The sol-gel was prepared with tetramethylorthosilicate (TMOS), as previously described [30,31]. Briefly, 3 mL of TMOS was hydrolyzed by 0.37 mL of 40 mM HCl in the presence of 2.5 mL MeOH at 60 °C for 1.5 h. This mixture was diluted with 200 µL of water and 1 mL of similarly hydrolyzed 3-aminopropyltrimethoxysilane (APTS). APTS introduces amino groups into the matrix and promoted sustained release of the enclosed contents in our previous studies [30]. The polymerization of this hydrolyzed TMOS was carried out at 40 °C. It formed a clear gel within 30 min.

#### 2.3.2. Preparation of NAC-SNO-np

Two grams of gel was mashed with a glass rod and cooled on ice. To this, 2 mL of freshly prepared, cold NAC-SNO (900 µmol) was added, which was prepared by mixing 900 µmol of nitrite and 1080 µmol of NAC. The excess NAC ensured the complete conversion of nitrite into SNO. This leaves an unreacted NAC of approximately 180 µmol in the mixture. The mixture was mixed on a lab rotator for 3 h at 4 °C. The mixture was then centrifuged at 1900× *g* for 5 min, supernatant separated, and particles lyophilized. The dried powder was ground into finer particles with a mortar and pestle (NAC-SNO-np). The NAC-SNO-nps were stored in aliquots at −80 °C. The final concentration of NAC-SNO in nanoparticles was 1.2 µmol/mg. The concentration of NAC in the particles was not determined. The particle size ranged from 200 to 2000 nm, as determined by dynamic light scattering.

### 2.4. Measuring the Release of NAC-SNO from NAC-SNO-np

NAC-SNO-nps were dispersed in phosphate-buffered saline (PBS) at 5 mg/mL and mixed on a lab rotator at room temperature. Aliquots were drawn at specific time intervals, diluted 10 times, and centrifuged at 17,000× *g* at 4 °C. The supernatants were separated and the absorbance at 335 nm was recorded on a spectrophotometer (Thermo Fisher Scientific, Waltham, MA, USA). Since *S*-nitrosothiols are temperature sensitive [32], the rate of decay of NAC-SNO at room temperature was also studied. NAC-SNO at comparable concentration to that in NAC-SNO-np was incubated in PBS at room temperature. Aliquots were diluted at specific time intervals and the absorbance at 335 nm was recorded.

### 2.5. Measuring the Release of NO from NAC-SNO-np

NO release from NAC-SNO-np and NAC-SNO was measured using a NO analyzer (Sievers 280i; GE Instruments, Boulder, CO, USA). NAC-SNO-np was dispersed in 5 mL of PBS at 5 mg/mL at room temperature. NAC-SNO at matching concentrations with NAC-SNO-np was used as a control. Release kinetics were analyzed using Sievers NO Analysis software (GE Instruments, Boulder, CO, USA) with 12 measurements per minute recorded for 280 min.

### 2.6. Planktonic and Biofilm Plate Preparation

In order to prepare *C. auris* for plating, logarithmically growing fungal cells prepared the previous night were collected by centrifugation, washed twice with PBS, counted using a hemocytometer, and suspended in a chemically defined minimal medium (20 mg/mL of thiamine, 30 mM glucose, 26 mM glycine, 20 mM MgSO4·7H2O, and 58.8 mM KH2PO4) supplemented with 5% fetal bovine serum (FBS; Atlanta Biologicals, Minneapolis, MN, USA) [25]. Two separate suspensions of *C. auris* were prepared, planktonic and biofilm. Planktonic cells were aspirated into 2 mL microcentifuge tubes where 100 µL of a 2 × 10^7^ cells/mL of fungal cell suspension were added to each tube. The planktonic tubes were then co-incubated with NAC-SNO-nps in a microtube rotator at 37 °C for 24 h. Biofilm preparations were plated onto a 96-well tissue culture treated microplate. The biofilm preparation was plated at a concentration of 1 × 10^7^ cells/mL, with 200 µL of fungal cell suspension in each well. The biofilm plate was incubated for 24 h to allow for mature biofilm formation prior to use in therapeutic testing.

### 2.7. Determining Susceptibility of C. auris to NAC-SNO-np

Optimal NAC-SNO-np dosage was determined by fungal cell growth curves performed on planktonic MMC 1 and MMC 2. Planktonic MMC strains were plated, as described above, at a concentration of 1 × 10^7^ cells/mL on a 96-well microplate and incubated with an additional 100 µL of increasing concentrations of NAC-SNO-np (1.25, 2.5, 5, and 10 mg/mL) suspended in control solution for 48 h. The optical density (OD) was measured at 492 nm in 24 h intervals. 

### 2.8. Measuring Planktonic and Biofilm Viability by CFU Killing Assay

Colony forming unit (CFU) killing assays were used to determine the susceptibility of *C. auris* strains to NAC-SNO-np. Three treatment solutions were used in this study: NAC-SNO-np, blank-np, and a control solution consisting of Minimal medium with 5% FBS. Blank-np consisted of the nanoparticle scaffolding without the addition of NAC or NAC-SNO. The 6 *C. auris* strains were grown as planktonic and biofilm forms and tested with these three solutions. For each experiment, treatment conditions were tested in triplicate. Each experiment was repeated in triplicate for every strain of *C. auris,* totaling 9 data points for each treatment group. 

Planktonic *C. auris* was grown, as described above, and 100 µL of fungal cell suspensions were incubated with a 100 µL solution of either 10 mg/mL NAC-SNO-np, 10 mg/mL blank-np, or control solution for 24 h. SAB agar plates were inoculated with 100 µL of a 10^6^ to 10^8^ times diluted solution from each treatment group and incubated for 48 h before CFUs were counted. Biofilm *C.* auris was grown as described above in 96 well plates. After 24 h of initial growth, the liquid medium was gently removed by pipette and 200 µL of a 10 mg/mL solution of NAC-SNO-np, 10 mg/mL blank-np, or a control solution were added followed by another 24 h incubation. After the incubation period, the fungal cells were collected by vigorous scraping and pipetting, and 100 µL of a 10^6^ to 10^8^ times diluted solution from each treatment group was then plated onto SAB agar plates, incubated for 48 h, and CFUs were counted.

### 2.9. Evaluating NAC-SNO-np on Biofilm Viability by Confocal Laser Scanning Microscopy

MMC 1 and MMC 2 biofilms were grown, as described above, except that 500 µL of a fungal cell suspension was inoculated into each chamber of an 8 well LabTek II Chambered #1.5 Coverglass System (Thermo Fisher Scientific, Waltham, MA, USA). Biofilms were incubated at 37 °C for 48 h, and the medium was replaced after 24 h. Both fungal strains were treated with a 10 mg/mL NAC-SNO-np treatment solution for 24 h. After treatment, biofilms were stained with LIVE/DEAD FungaLight (Thermo Fisher Scientific, Waltham, MA, USA). Images of biofilms were obtained using a TCS SP5 Confocal Laser Scanning Microscope (Leica Microsystems, Wetzlar, Germany) configured to a 405 nm diode and 488 nm argon laser with a 63× objective. Five Z-stacks of each individual sample were taken corresponding to the areas of highest biofilm growth. Each Z-stack consisted of 0.75 μm individually sliced images of the same area of biofilm. Volocity software (Version 6.5.1, PerkinElmer, Waltham, MA, USA) was used to quantify biofilm viability and biomass within each Z-stack. This experiment was performed in triplicate for both strains.

### 2.10. Evaluating Blank-np Penetration by Confocal Microscopy

To evaluate the ability of blank-np to penetrate through *C. auris* biofilms, MMC 1 and MMC 2 biofilms were grown in an 8 well chamber, as described above. After 48 h of growth, each biofilm was stained with calcofluor white stain to visualize the fungal cell wall (Eng. Scientific, Inc. Clifton, NJ, USA) and treated with 200 µL of a 1 mg/mL Syto-9-stained blank-np solution for 24 h. Biofilms were then imaged using a TCS SP5 Confocal Laser Scanning Microscope with the same configurations as above. Three Z-stacks (0.75 µm individual sliced images) were taken from each well. Volocity software was used to evaluate Blank-np biofilm penetration. 

### 2.11. Statistical Analysis

Statistical analysis was performed using GraphPad Prism (Version 8.3, GraphPad Software, La Jolla, San Diego, CA, USA). One-way ANOVA, and independent t-tests were used to analyze differences between CFU treatment groups and biofilm volume, respectively. Statistical significance was set at the standard *p* < 0.05.

## 3. Results

### 3.1. NAC-SNO-np NAC-SNO Release Curve

**Figure 1:**
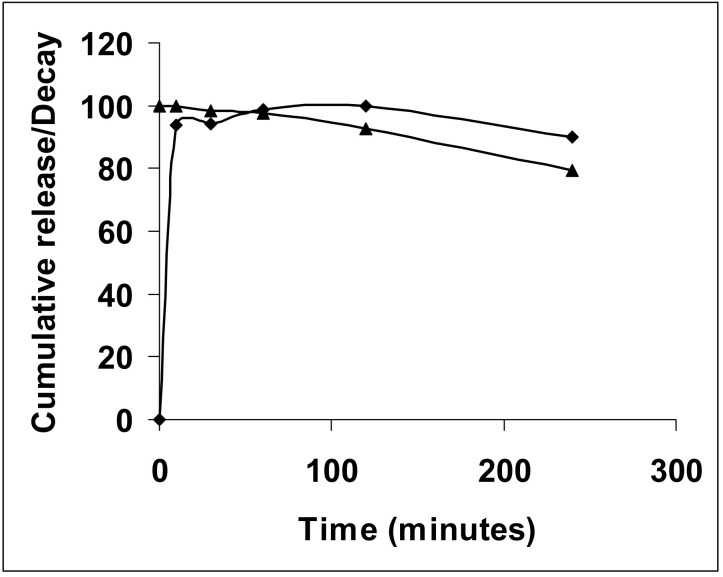
Rate of NAC-SNO release from nanoparticles; NAC-SNO-nps were dispersed in PBS, pH 7.4 at room temperature. The amount of NAC-SNO released (diamond) was determined from the absorbance of the buffer at 335 nm. The rate of decay of NAC-SNO (triangle) at room temperature is also shown.

The release rate of NAC-SNO from NAC-SNO-np was studied at room temperature. Only 50% of the total amount of enclosed NAC-SNO released in the first 4 h. The maximum amount released in these 4 h is taken as the total amount to calculate the cumulative release, as the decay of NAC-SNO disrupted further measurements. As can be seen in Figure 1, more than 90% of NAC-SNO was released instantaneously followed by a very slow release. The initial rapid release may be due to the release from the surface of the nanoparticles, and the contents contained within the particles are released at a slower rate. The decline in the curve with time is an indication of dissociation of NAC-SNO at room temperature. This decline is more prominent at later time points, due to which the released amounts are underestimated. 

### 3.2. NAC-SNO-np NO Release Curve

**Figure 2:**
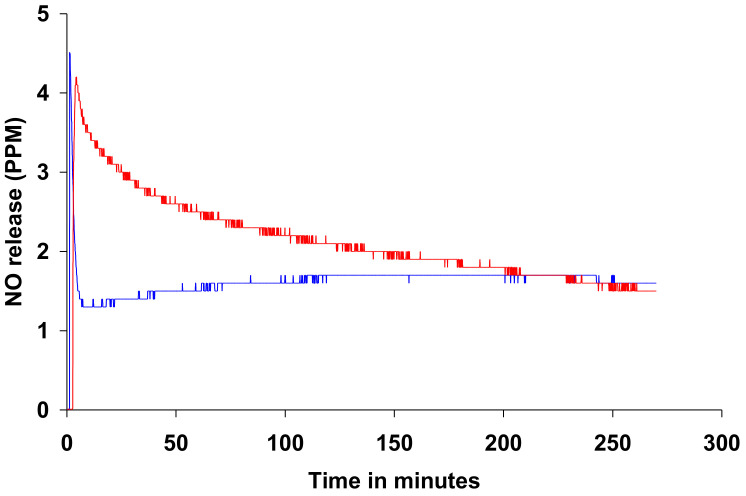
Rate of nitric oxide release from NAC-SNO-np, as monitored by a NO analyzer; NAC-SNO-np (red) or NAC-SNO (blue) was added to PBS, pH 7.4, at room temperature.

The rate of NO release from NAC-SNO-np in PBS, pH 7.4, was studied at room temperature using a NO analyzer (Figure 2). NAC-SNO-np initially released NO approximately 4 ppm and exhibited a gradual decrease in rate to 1.5 ppm by 280 min. This profile matches the rate of NAC-SNO release from the particles—A rapid release followed by a steady, slow release (Figure 1). NAC-SNO released an initial bolus of NO (approximately 4.5 ppm) and dropped to 1.3 ppm within 5 min. After, a slow increase followed that equilibrated at a steady release rate of 1.7 ppm, which was observed until the end of the experiment 280 min later.

### 3.3. Spectrophotometric Assay

**Figure 3:**
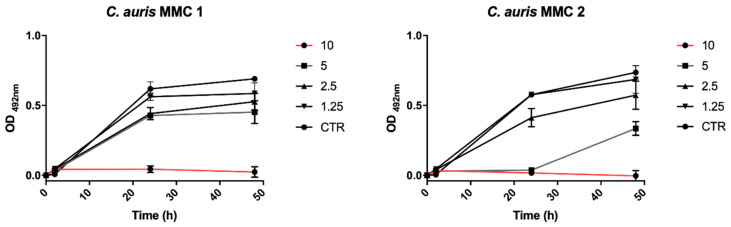
Dose-dependent growth curves were generated for planktonic *C. auris* MMC 1 and MMC 2 to determine the minimum concentration of NAC-SNO-np to eradicate fungal cells.

Spectrophotometric assay of MMC 1 and MMC 2 treated with NAC-SNO-np demonstrated complete inhibition of fungal cell growth under planktonic conditions for both strains at a final NAC-SNO-np concentration of 10 mg/mL (Figure 3). Dose-dependent growth curves were performed in triplicate. NAC-SNO-np decreased *C. auris* growth in a dose-dependent manner, with a final concentration of 10 mg/mL NAC-SNO-np sufficient to arrest planktonic *C. auris*. As a result, 10 mg/mL was chosen as the appropriate NAC-SNO-np concentration for the subsequent experiments in this study. 

### 3.4. CFU Killing Assay

**Figure 4:**
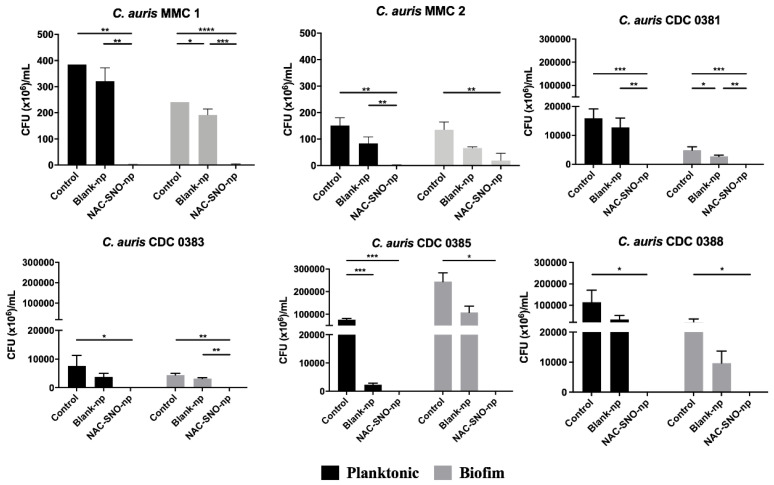
Planktonic and biofilm *C. auris* were incubated for 48 h and treated with NAC-SNO-np (10 mg/mL). CFUs were counted after 24 h. Statistical analysis was performed by one-way Anova with Tukey’s multiple comparison test. * *p* < 0.05, ** *p* < 0.01, *** *p* < 0.001, **** *p* < 0.0001.

Planktonic and biofilm CFU killing assays were performed for the six strains of *C. auris* (Figure 4). Varying rates of colony formation were seen between each strain of *C. auris*, with the CDC strains forming many more colonies than their MMC counterparts. With the exception of the biofilm preparation of MMC 2, each strain treated with NAC-SNO-np demonstrated a significant reduction for both planktonic and biofilm *C. auris* compared to control conditions (*p* < 0.05). Specifically, planktonic MMC 1 had a 9.59 log decrease and biofilm MMC 1 had a 9.38 log decrease. Planktonic and biofilm CDC 0381 had a 10.2 and 9.68 log reduction in CFUs, respectively, while planktonic and biofilm CDC 0383 CFUs had a 9.88 log reduction and a 2.65 log reduction, respectively. Planktonic CDC 0385 had a 3.13 log reduction and the biofilm CFUs had a 2.21 log reduction. Finally, planktonic and biofilm CDC 0388 had a 1.49 and 3.04 log reduction, respectively. MMC 2 planktonic plates had a 9.18 log reduction, and biofilm plates had a 0.98 log reduction, remaining statistically significant (*p* < 0.01). Across the six strains, there was a non-significant reduction in planktonic CFU count between the control group and the blank-np treatment group, except for CDC 0385, where the planktonic colonies demonstrated a significant decrease in fungal growth due to blank-np (*p* < 0.001). Furthermore, for the biofilm colonies, MMC 1 and CDC 0381 showed a significant decrease in CFUs (*p* < 0.05). 

### 3.5. Biofilm Viability by Confocal Microscopy

**Figure 5:**
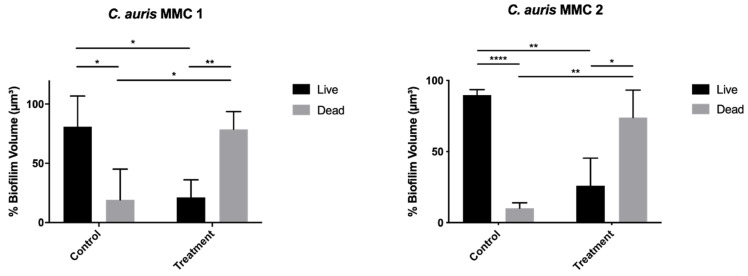
*C. auris* biofilms were grown for 48 h and treated with 500 µL of NAC-SNO-np (10 mg/mL) for 24 h. Graphs represent averages and standard deviations. Statistical analysis was performed by independent t-tests. * p < 0.05, ** p < 0.01, and **** p < 0.0001.

**Figure 6:**
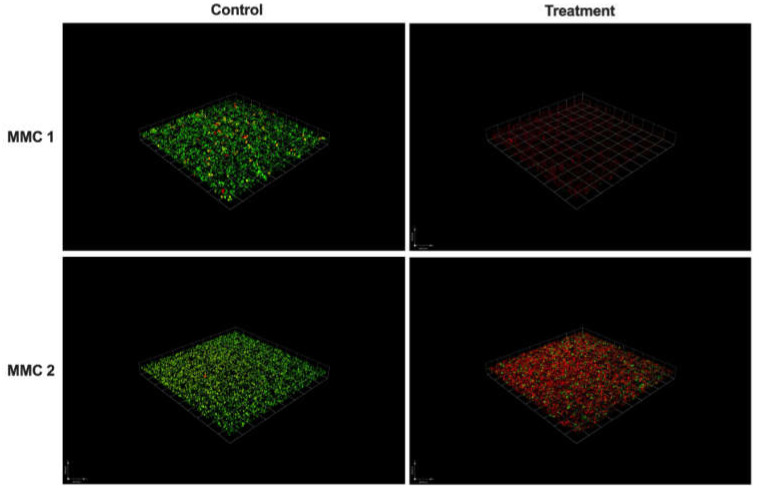
*C. auris* biofilms grown for 48 h and treated with 500 µL of NAC-SNO-np (10 mg/mL) for 24 h. *C. auris* biofilm stained with FungaLight (live: green; dead: red). Scale bar represents 100 µm.

For both MMC 1 and MMC 2, control wells had 80 and 90% viability, respectively, as measured by confocal microscopy biofilm volume and viability staining (Figure 5 and Figure 6). After a 24 h treatment with a 10 mg/mL NAC-SNO-np solution, both strains demonstrated significant susceptibility to the NAC-SNO-np treatment. MMC 1 showed a 74% reduction in biofilm viability, which corresponds to a statistically significant 0.58 log reduction (*p* < 0.05; Figure 6). Compared to MMC 1, the MMC 2 biofilm was slightly less susceptible to NAC-SNO-np treatment, but still demonstrated a statistically significant 71% decrease or 0.54 log reduction in biofilm viability (*p* < 0.01). 

### 3.6. Blank-np Biofilm Penetration

**Figure 7:**
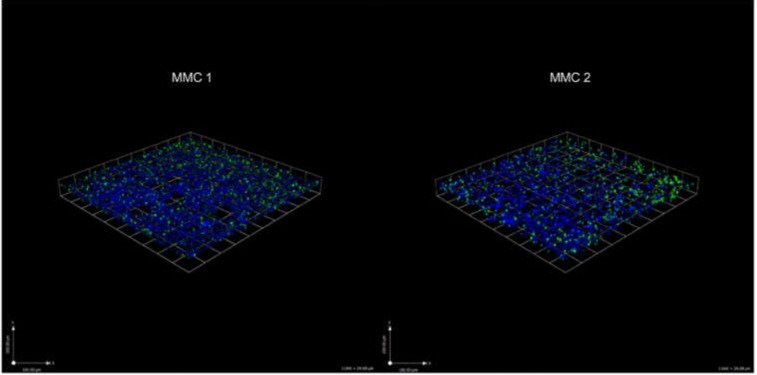
*C. auris* biofilms grown for 48 h and treated with 200 µL of blank-np (1 mg/mL) for 24 h. *C. auris* biofilm stained with calcoflour (cell wall: blue) and Syto-9 (blank-np: green). Scale bar represents 100 µm.

Confocal microscopy imaging of MMC 1 and MMC 2 biofilms visually shows homogenous distribution of the particles within the wells with little clumping and demonstrates effective penetration of our nanoparticles (Figure 7).

## 4. Discussion

Biofilms are a principal form of microbial growth [33]. They are critical to the development of certain infections, especially in the presence of foreign material such as indwelling catheters. Fungal cells in biofilms are more resistant to antifungal drugs than planktonic cells. Biofilms are complex surface-associated cell populations embedded in an extracellular matrix (ECM) that possess distinct phenotypes compared to their planktonic cell counterparts [33]. Other contributing factors include metabolic heterogeneity intrinsic to biofilms, biofilm-associated upregulation of efflux pump genes, nutrients, quorum-sensing molecules, and surface contact. The actual fold increase in resistance varies with both the drug and species. *Candida albicans* and *Candida parapsilosis* biofilms are relatively resistant to fluconazole, amphotericin B, nystatin, voriconazole, and others [33], and it appears that *C. auris* has many of the same mechanisms, particularly the efflux pump, that contribute to the notorious recalcitrance of its biofilm to common antifungal medications [34]. 

*C. auris* is an emerging fungal pathogen that has garnered attention due to its widespread antifungal resistance and high mortality rates. In the present work, we demonstrate the efficacy of a novel NAC-SNO-np treatment platform against six strains of both planktonic and biofilm forms of *C. auris*. This nanoparticle formulation releases NO, NAC, and NAC-SNO—All of which have previously demonstrated antimicrobial effects [35,36,37,38]. Thiol groups from NAC have been shown to exhibit antimicrobial activity through the formation of peroxynitrite [39]. The antibacterial activity of S-nitrosothiols is attributed to their capability of modifying thiols on enzymes by transnitrosation [40]. Figure 2 portrays the similar initial peak of NO release in both the NAC-SNO-np and NAC-SNO solution. Thereafter, we demonstrate the capability of NAC-SNO-nps to slowly release NO over a sustained period of time compared to the NAC-SNO solution. This slow release allows for NO to be constantly delivered to the *C. auris* planktonic cells and biofilm. 

Our previous work has shown that the nanoparticle delivery platform is a viable way of delivering NO, and delivery of these nanoparticles had substantial antibacterial and antifungal activity against both planktonic and biofilm forms [31,41,42]. This is particularly useful when considering that *C. auris* colonizes the axilla, groin, and difficult to reach areas like the ear and nose where topical administration can be effective [14]. This present study demonstrates that NAC-SNO is an effective compound for eradicating *C. auris* biofilms. 

The spectrophotometric assay identified a dose of 10 mg/mL that was used throughout this study. This dose was also used in our past studies, as it was efficacious on bacterial species [42]. The CFU killing assays highlight the efficacy of NAC-SNO-np. NAC-SNO-np completely eradicated planktonic *C. auris* in all six strains. In our biofilm CFU assays, NAC-SNO-np also completely eradicated *C. auris* in the CDC strains, and resulted in growth reductions of 98% and 75% for MMC 1 and MMC 2 strains, respectively. Interestingly, it appears that there is no correlation in susceptibility of planktonic or biofilm *C. auris* with known resistance to antifungal drugs, a positive sign for the increasing resistance to antifungals. Moreover, *C. auris* grew efficiently in the 8-well chamber, and NAC-SNO-np had significant antibiofilm activity against MMC 1 and MMC 2. Confocal laser scanning microscopy corroborates this result visually as fungal cells were fewer and appeared disrupted. This indicates potent susceptibility to NAC-SNO-np. However, this decrease did not reach the 99% reduction that is considered fungicidal [43]. Despite the broad differences in susceptibilities to standard antifungals (Table 1), each *C. auris* strain was susceptible to the NAC-SNO-np under planktonic and biofilm growth conditions. This is a promising sign for NAC-SNO-np and NO-releasing therapeutics as a future treatment option for resistant species.

To further examine the efficacy of our nanoparticle delivery platform on *C. auris* biofilm, we incubated blank-np nanoparticles with *C. auris*. We have noted that our blank-np appears to have some significant effects on *C. auris* in our CFU killing assay for both the planktonic and biofilm forms. Our findings illustrate the capability of nanoparticles to efficiently penetrate *C. auris* biofilm, and we can infer from our results that the nanoparticles may inhibit biofilm growth (Figure 5). This is an interesting finding that suggests the nanoparticle scaffolding itself may inhibit the growth of *C. auris.* These effects may be due to the physico-chemical properties of the nanoparticles (e.g., particle size, charge, and surface chemistry) causing disruption in cell-to-cell communication and other cellular processes such as quorum sensing that are involved in biofilm formation [31]. We have found similar results in our previous studies with different blank particle formulations [31,41], where thiols are known to interact with disulfide bonds and react with extracellular polysaccharides, impairing ECM formation and adhesion of biofilm to surfaces [35,36]. In the blank-np for this experiment, the amines present on the particles may also have contributed to the antimicrobial activity of the particles [44]. While the physical presence of the blank nanoparticles may have an effect of the biofilm, the addition of NAC-SNO dramatically changes the effect of nanoparticles in the biofilm environment. 

Limitations of this study include the inability to temperature regulate our NO analyzer to run at 37 °C, which resulted in our testing the materials at a uniform 30 °C. However, both NO and NAC-SNO release was run at the same temperature for consistency. Other limitations are the financial and time constraints related to CLSM, precluding testing on CDC strains of *C. auris*. However, we would expect a similar pattern of biofilm disruption from NAC-SNO-np, as demonstrated by our CFU killing assay on these strains. Other fungal strains may or may not be similarly affected by NAC-SNO-np, and future studies will need to focus on the antimicrobial effects of NAC-SNO on other fungal and bacterial species as well as investigate any cellular toxicity. 

## 5. Conclusions

NAC-SNO-np effectively eradicates planktonic *C. auris* growth and significantly reduces and disrupts biofilm growth. NAC-SNO-np shows great promise as a future prophylactic and therapeutic for *C. auris* and other drug-resistant pathogens.

## Figures and Tables

**Table 1:** *C. auris* strains were collected from Montefiore Medical Center (MMC) and the Centers for Disease Control (CDC). Tentative MIC breakpoints (bolded values) for *C. auris* are reported from the CDC as: amphotericin B ≥ 2; caspofungin ≥ 2; fluconazole ≥ 32.

Fungal Isolate	Origin	MIC (μg/mL)		
		Amphotericin B	Caspofungin	Fluconazole
MMC 1	Bronx, NY	1.6	2	>256 ^a^
MMC 2	Bronx, NY	0.8	1.6	8
CDC 0381	Japan	0.38	0.125	4
CDC 0383	South Africa	0.38	**16**	**128**
CDC 0385	Venezuela	0.5	0.5	>**256**
CDC 0388	Pakistan	1.5	0.5	**256**

^a^ MMC1 was resistant to fluconazole at a concentration of 1000 µg/mL.
